# Task Dependency of Grip Stiffness—A Study of Human Grip Force and Grip Stiffness Dependency during Two Different Tasks with Same Grip Forces

**DOI:** 10.1371/journal.pone.0080889

**Published:** 2013-12-04

**Authors:** Hannes Höppner, Joseph McIntyre, Patrick van der Smagt

**Affiliations:** 1 Institute of Robotics and Mechatronics, German Aerospace Center, Wessling, Germany; 2 Centre d'Etudes de la Sensorimotricité, Centre National de la Recherche Scientifique and Université Paris Descartes, Paris, France; 3 Faculty for Informatics, Technische Universität München, Munich, Germany; University of California Merced, United States of America

## Abstract

It is widely known that the pinch-grip forces of the human hand are linearly related to the weight of the grasped object. Less is known about the relationship between grip force and grip *stiffness*. We set out to determine variations to these dependencies in different tasks with and without visual feedback. In two different settings, subjects were asked to (a) grasp and hold a stiffness-measuring manipulandum with a predefined grip force, differing from experiment to experiment, or (b) grasp and hold this manipulandum of which we varied the weight between trials in a more natural task. Both situations led to grip forces in comparable ranges. As the measured grip stiffness is the result of muscle and tendon properties, and since muscle/tendon stiffness increases more-or-less linearly as a function of muscle force, we found, as might be predicted, a linear relationship between grip force and grip stiffness. However, the measured stiffness ranges and the increase of stiffness with grip force varied significantly between the two tasks. Furthermore, we found a strong correlation between regression slope and mean stiffness for the force task which we ascribe to a force stiffness curve going through the origin. Based on a biomechanical model, we attributed the difference between both tasks to changes in wrist configuration, rather than to changes in cocontraction. In a new set of experiments where we prevent the wrist from moving by fixing it and resting it on a pedestal, we found subjects exhibiting similar stiffness/force characteristics in both tasks.

## Introduction

A vast body of literature is devoted to the regulation of grip force. Indeed, the force necessary to stably hold an object in our hand is continuously regulated by the CNS [1], [2] in a process known as “grip-force/load-force coupling”. Already in children at the age of 2, this grip force is regulated depending on an object's weight [3]. Furthermore, the CNS is capable of modulating grip force to account for load forces acting on the hand-held object, such as the inertial forces induced by movement of the arm [4], [5], or whole body movement during running [6] and jumping [7].

It has been shown that forces of an uncompensated grip decreases for contracting and increases for expanding objects [8], which evokes the concept of grip *stiffness* (i.e., the change in grip force versus a change in grip aperture) and may play an important role in maintaining grip stability. In a recent study [9] we measured grip stiffness as a function of grip force applied to an object held in a pinch grip. Participants were instructed to perform a force task with visual feedback, i.e., exert a predefined force which could be monitored by displaying the exerted force measured with a load cell. By applying very fast finger position perturbations during grip, we measured the part of stiffness that is related to biomechanics only, known as *passive intrinsic stiffness*, excluding influences from proprioceptive feedback. With these experiments, we demonstrated a linear relationship between grip force and intrinsic grip stiffness contributed by the passive properties of the corresponding muscles. We further showed that this conforms to a model of the pinching hand in which muscle exhibit elastic properties that can be represented by (nonlinear) exponential force-generating elements. A number of studies confirm this finding of a monotonic increase of finger force or torque with stiffness[10]–[13].

But how do load force, grip force and grip stiffness relate to each other? Can grip stiffness be modulated independent of grip force and if so, would such modulation have functional significance? Furthermore, the way in which we required subjects to apply different grip forces in our previous study (i.e., through visual feedback) was not very natural. What would the stiffnesses be like if a subject would lift an object in a weight task without any feedback about the applied force? Would the stiffnesses measured in the two tasks be comparable? Or would subjects be able to regulate force and stiffness independently?

Two possibilities to decouple grip stiffness and force are acknowledged: either by cocontraction of antagonistic pairs of muscles or by changing the finger/wrist configuration. Carter *et al.* showed that for zero net torque at the interphalangeal joint in the human thumb, joint stiffness highly increases with cocontraction. This demonstrates that net torque or force alone does not determine joint stiffness [13]. Furthermore, wrist flexion and extension causes stretching and shortening of, among others, the corresponding flexor digitorum superficialis and profundus muscles [14], affecting their force/activation relationship as described in the Hill muscle model [15]. This effect can reduce the maximum grip force to 73% of its maximum [16]. Thus, changes in wrist configuration should lead to changes in both grip force and grip stiffness. But even during wrist movements of up to 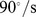, the CNS is able to keep grip force stable [2].

Does the neuromuscular system allow for an active use of these effects on grip stiffness and an independent control of both, force and stiffness? White *et al.* showed that the CNS is able to decouple grip and load force in anticipation of a collision, with a rise in grip force before the expected impact and a peak in grip force around 65 ms after the impact [17]. They hypothesized that the CNS increases the net grip force in order regulate grip stiffness and damping, with the goal of optimizing stability in object manipulation. They did not, however, directly measure grip stiffness. Furthermore, not only might one wish to modulate the grip force on an object to keep it stable in the face of predictable events like a self-generated collision, one might also wish to regulate the stiffness of the grip in anticipation of unexpected perturbations depending on the constraints of a specific task.

In the current study we set out to test for grip stiffness modulation as a function of the natural tendency to increase grip force when lifting increasingly heavy objects [1], [2]. We asked human participants to perform the visually-guided force-control task (FT) described above and a task in which they lifted objects of different weight (WT), without any specific instructions or visual feedback about the forces applied to the object in the pinch grip. Given our previous conjecture for the force task:
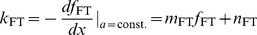
(1)where 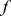 is the exerted force, 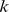 the stiffness of the pinch grip, 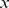 the pinch grip aperture, 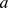 the muscle activation and 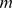 and 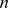 are slope [1/m] and offset [N/m], we can similarly conjecture
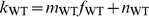
(2)for the weight task. To compare grip-stiffness/grip-force coupling between these two tasks, we set out to test the following specific hypotheses:

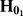
: The measured stiffness in both tasks are equal for same grip force, i.e. 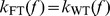;

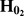
: The relationship between grip stiffness and grip force are equal for the two tasks, i.e. 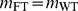 and 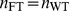.

### General model of the fingers

In this section we will introduce a general finger model which describes the influence of cocontraction and kinematics on grip stiffness. The model will help us to interpret the measurements. Eq. (3) describes the restoring finger forces 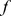 and their relation to the Cartesian stiffness matrix 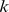. This stiffness matrix describes the elastic behaviour caused by a displacement 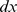 of the end point of a finger. Eq. (4) does the same for the finger joint torques 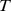 vs. joint stiffness 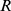 which is caused by an angular displacement 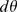. Finally, Eq. (5) describes the relation between muscle forces 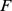, muscle stiffness matrix 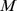, and muscle elongation 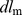:
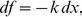
(3)

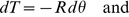
(4)

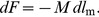
(5)ignoretrue The finger and joint velocities are coupled by the Jacobian matrix 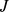, taking into account finger phalanx lengths; while muscle and joint velocities are coupled by the Jacobian matrix 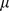, which corresponds to muscles moment arms:
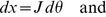
(6)

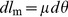
(7)ignoretrue from which we can derive that
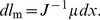
(8)


Note that 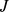 and 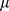 are, in fact, functions of 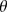, but for readability we leave this out in our notation. Eq. (8) couples the finger endpoint and muscle displacements via a ratio of the two Jacobians.

The joint torque is coupled to the Cartesian and muscle force similar to the velocities by the kinematic chain but also by the moment arms represented by the Jacobian matrices:
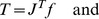
(9)

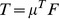
(10)ignoretrue from which we find
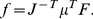
(11)


Again, the endpoint and muscle forces are coupled via a ratio of the two Jacobians. Using these equations and the assumption that the two Jacobians do not change for incremental angular displacements, the relations between the different stiffnesses become
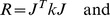
(12)

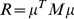
(13)ignoretrue leading to
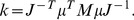
(14)


**Figure 1:**
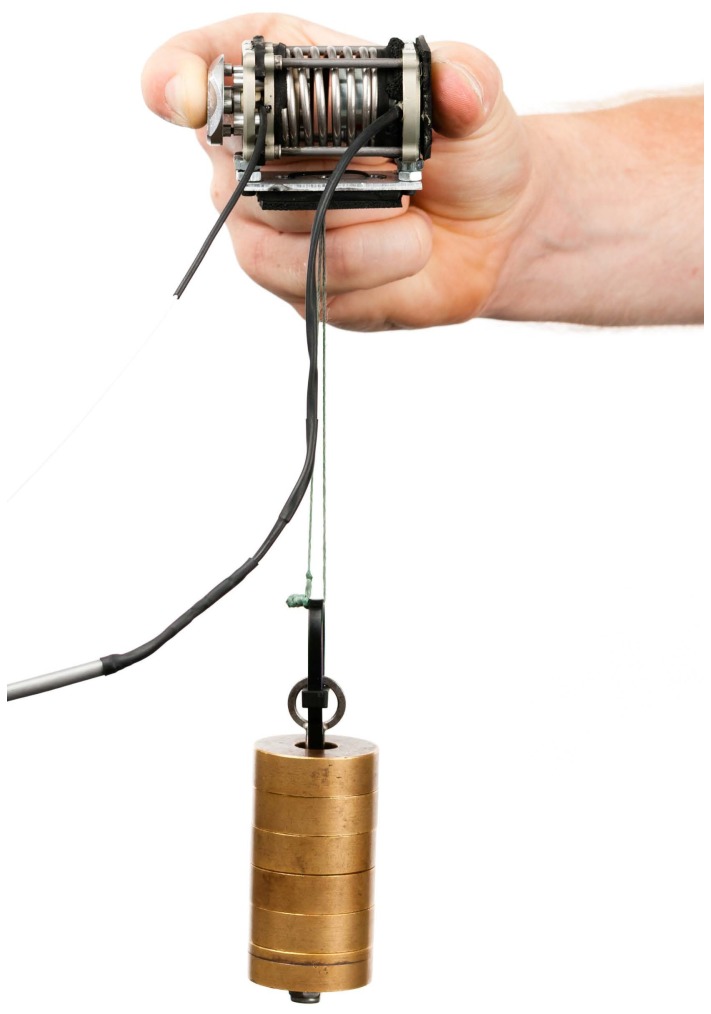
Stiffness change due to cocontraction. Increasing the cocontraction of the corresponding muscles increases the offset in the stiffness/force relationship.

Combining Eqs. (11) and (14) we conclude that, if 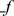 and 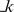 are linearly related, then so are 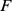 and 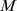. In words: if the grasp force and Cartesian stiffness are linearly dependent, then this is caused by a linear relationship between the muscle force and muscle stiffness. From these equations we can derive the influence of the two strategies for changing endpoint stiffness and its effects on the stiffness/force characteristic:

Cocontraction will increase the force and stiffness of agonist and antagonist muscles within each finger, while maintaining the same net force 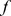 applied by the finger tips. For a given level of co-activation, the operating point on the stiffness/force characteristic of each muscle and each joint will change, but the slope of the stiffness/force characteristic will not be affected. Under the assumption that the kinematic configuration will not be changed due to increased internal forces, cocontraction will lead to an increase in the muscle stiffness 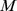 and a proportional increase of the Cartesian stiffness 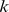, without a change of grip force. Thus changing stiffness caused by *cocontraction will affect the offset, but not the slope* of the (linear) grip force–grip stiffness relation. This principle is illustrated in Fig. 1.

From Eq. (14) we can see that a change in the Jacobian by, e.g., changing finger or wrist orientation will have a nonlinear (quadratic) effect on the stiffness/force characteristic and will affect slope and offset.

Note that both strategies will not affect the linear relation between force and stiffness while cocontraction and kinematic orientation remain the same. A change of the slope between the two tasks will indicate an influence caused by a change of the Jacobian rather than by cocontraction. A change of the offset, however, can be caused by either or both. Furthermore, a change in the kinematic configuration possibly predominates effects of cocontraction on the stiffness/force characteristics.

## Methods

### Device description

**Figure 2:**
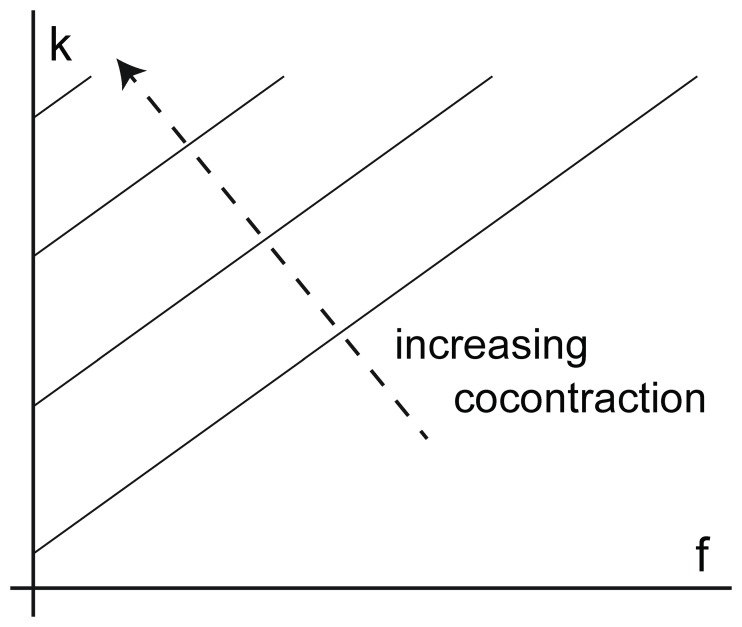
Cross sectional view of the Grasp Perturbator.

The Grasp Perturbator (Fig. 2) used in this experiment was a small cylindrical device with which finger stiffness during pinch grip *in flexion* can be identified. A spring was preloaded by an electromagnet (blue) fixed to a frame (gray) that holds a moving part (red). The grip force was measured with a load cell (black). Note that this device could only apply perturbations in one direction, measuring stiffness of an expanding object. Precise grip force measurement was obtained by guiding the grip force through a button (green) to the load cell. Releasing the spring caused the device to elongate by 7 mm within a few ms. The Perturbator weighed 187 g and its length expanded from 57 to 64 mm when activated. As noted by Van Doren [10], grasp span has a small effect on grip stiffness (stiffness changed only 5% for a change of 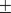 2 cm in grasp span); we therefore have chosen a fixed Perturbator size. The spring force was 140 N when loaded and 100 N when unloaded, i.e., considerably higher than human pinch-grip force, ensuring identical experimental conditions independent of how firmly the Perturbator was held.

The measurement setup consisted of a host running Windows and a real-time target machine running QNX. The real-time machine ran a Matlab/Simulink model to control the electromagnet and read out the force sensor at 10 kHz. After pressing a release button, the perturbation was applied after a random delay 4 to 7 s. The load cell consisted of a KM10 force sensor and a measurement amplifier GSV-11H (both from ME-Messsysteme GmbH) with a nominal force of 100 N and an overall accuracy of the force signal of 0.1 N were used. We verified that perturbations caused no significant phantom force changes in the device by testing it with known springs. The offset of the measured force signal was calibrated before each trial.

In this study we focused on influences of underlying (bio)mechanical principles, rather than of reflexive feedback, and measured the combination of passive (surrounding tissue and ligaments) and intrinsic stiffness only (stiffness which can be attributed to the active muscle fibres); we refrained from measuring influences of reflexive stiffness. By measuring the grip force with an internal load cell before and shortly after the perturbation—well before the effects of short-latency reflexes—the grip stiffness contributed by the intrinsic properties of the muscles could be identified.

### Experimental procedure

A total of 15 healthy right-handed male subjects, age 22–45 years, performed the two experimental protocols, WT and FT, as described below. No subject had a history of neurological disorder nor neuromuscular injury affecting the CNS or the muscles. All subjects gave written consent to the procedures which were conducted partially in accordance with the principles of the Helsinki agreement. Non-conformity concerns the point B-16 of the 59th World Medical Association Declaration of Helsinki, Seoul, October 2008: no physician has supervised the experiment. The collection of subject data was approved by the institutional board for protection of data privacy and by the work council of the German Aerospace Center.

All subjects had previous experience working with the Perturbator and were able to stably hold the device, even after the perturbation. Fully naive subjects would often drop the device during perturbation, leading to useless data because of a missing second static force level.

Ten subjects performed the main experiment in which the arm and wrist were free to move, although subjects were instructed to hold the forearm steady in a horizontal posture. These ten subjects were divided into two groups, counter-balanced as to whether FT or WT was done first (E1 and E2, respectively). To investigate whether changes in the wrist configuration might have an influence on grip stiffness an additional group of 6 subjects (E3) performed the two protocols with the wrist held at a constant orientation with respect to the forearm and with the relaxed arm and wrist supported by a table. We favored fixation over controlling wrist position using optical tracking in order to keep the task natural and to avoid providing visual feedback in the WT. Half of the subjects in E3 (subjects S11, S12, S13) did WT first, the rest FT first. Note that one subject took part in two experiments, and is referred to as S6-1 in E2 and S6-2 in E3. The whole experiment lasted about 90 minutes per subject. No subject reported discomfort during FT, some reported fatigue during WT. Subjects stood throughout the experiment, except for a 10-minute break between FT and WT. We found standing and lifting weights with respect to the WT intuitively more natural than sitting.

### Weight Task

**Figure 3:**
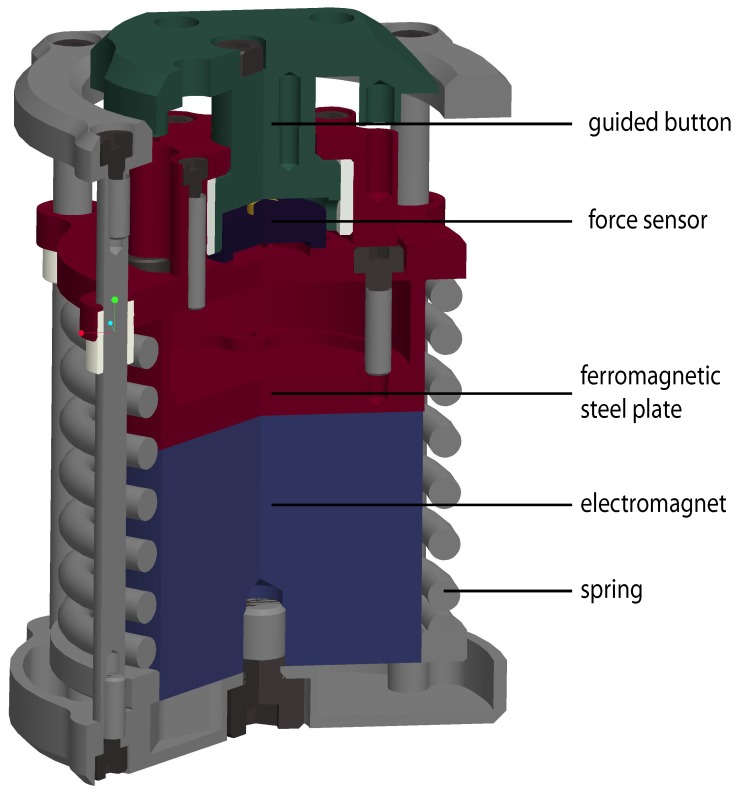
Grasp Perturbator held in a pinch grasp with attached weights.

In the WT, six different weights of from 0.2, 0.4, 1.2 kg were attached to the Perturbator, and for 10 trials each the subjects had to lift the device off of the table (Fig. 3). The lower arm was requested to be held at approximately a 90 degree angle with the body. There was no visual feedback w.r.t. the grip force to the subject. Once the grasp was stable, the experimenter pressed the button to apply the perturbation between 4 and 7 s later (randomly chosen by the control computer).

### Force Task

The procedure of the FT was almost the same as in our previous study [9], except that the subjects lifted and held the Perturbator above the table (as in WT, but with no additional weight attached). The subject received visual feedback about the actual force applied to the Perturbator and was asked to maintain a visually instructed predefined force. Once this force level was reached, the release button was pressed by the experimenter, unknown to the subject, and the perturbation was performed between 4 and 7 s later. Six instructed force levels were randomly presented to each subject, for a total of 10 times per force level.

Since applied grip forces for lifting the weights differed considerably across subjects, we measured the natural grip force when holding the device with different weights—thus leading to different grip forces—and chose 6 different grip force levels to subsequently use for FT. If subjects were instructed to do the FT first, we asked them to lift the Perturbator once with each weight attached before the FT without applying any perturbations. If the WT had to be done first, we used the information of the WT to estimate the required force levels for the FT.

For both tasks, we preferred experimenter release over automated release because previous experiments revealed increased participant fatigue in the latter case—holding the force level steady for a while, especially at high levels is increasingly difficult and troublesome. Note that the non-rigid coupling between the Perturbator and the additional weight in the WT meant that the inertia of the Perturbator was effectively constant, so that this effect did not have to be accounted for in the data analysis.

### Data processing

**Figure 4:**
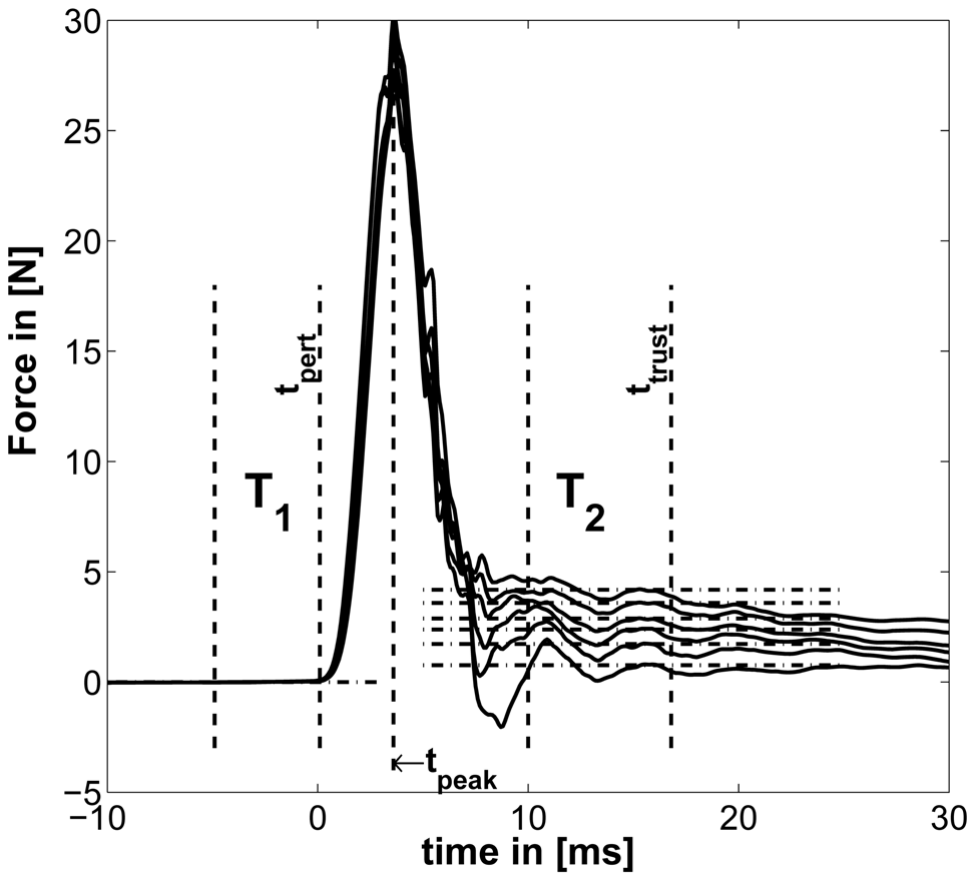
Example for typical Perturbation profile. Force profile before, during, and after a perturbation starting at 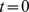. Additionally, the time windows 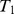 and 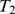 and the mean of force for six force levels are depicted (mean force 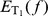 subtracted). The length of 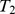 and 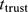 were found to be optimal at 6.8 and 16.7 ms, respectively.

The force signals were first filtered using a 21-point moving average filter. The time when the electromagnet was released and the time 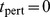 at which the perturbation started (see Fig. 4) varied considerably because the breakdown of the electromagnetic field depends highly on the applied grip forces. We therefore defined 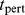 as the end of the period 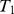, where 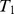 was defined as the last 10 ms time interval before 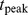 having a standard deviation below 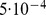 N. These numbers were empirically determined and led to stable results. The rise time between start of the perturbation and 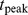 was on average 3.6 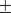 0.62 ms (SD) and showed no significant correlation with force and weight levels.

The time interval 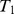 was used to determine the force level before the perturbation; conversely, we needed to determine a time window 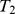 over which the force level *after* perturbation was computed. We defined a second time 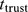 within which one can ignore effects of fast reflex responses and trust the data to be purely intrinsic, in order to measure tendon- and muscle-based influences only. The mean onset latency of the short-latency reflex is about 30.7 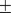 1.7 ms (SD) for the first dorsal interosseus in the hand [18]. In [19] Allum *et al.* reported a delay of about 20 ms between the onset of the short-latency reflex and first measurable changes in muscle force of stretching triceps surae muscles and releasing tibialis anterior muscles elicited by electrical stimulation. Thus, assuming that this feedback does not have a measurable influence within 40 ms, 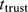 was allowed to vary downward from 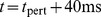 and the duration 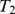 was allowed to vary between 5 and 20 ms so as to minimize an objective function.

We decided to use one time window 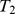 for all trials with a fixed start and end rather than optimizing either subject-wise or even force level-wise. This avoided comparing different lumped stiffnesses that are affected variously by damping and inertia, even if their influence was expected to be small. Furthermore, we looked at the gradient of the calculated stiffness values normalized by their mean as a kind of measure for stability of the achieved results. The data showed that stable results were achieved if the end of the second time window 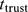 was at least 4 ms higher than the length of the time window 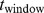. This corresponded to a second time window not intersecting the first and left out the peak of perturbation (see Fig. 4). Thus, 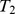 was varied so as to optimize the objective function
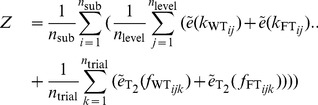
(15)using the whole number of trials 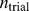, levels 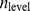 and subjects 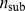. We introduce the CSE 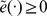


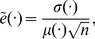
(16)which combines the coefficient of variation and standard error. The standard error compensates the standard deviation 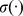 for sample size 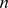 assessing low sample sizes with a higher standard error; the coefficient of variation is a normalized measure of the standard deviation and compensates for the sample mean 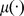. Since the objective function Eq. (15) mixes data sets of different size and from different dimensions, we had to compensate the standard deviation 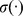 for both. 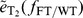 denotes the CSE of force for each trial within 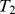 and 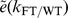 represents CSE of stiffness values for the different trials, for FT and for WT, respectively. The objective of this optimization was to minimize the oscillations within time interval 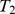 and the variation of resulting stiffness values measured under *exactly* the same conditions. Note, that we minimized the variation of stiffness between experiments with identical conditions rather than between different stiffnesses, grip forces, or subjects. The stiffness of each trial was calculated using
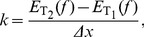
(17)where 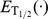 denotes the average over time intervals 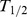. The length of the second time interval 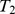 and its end 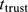 were found to be optimal under named constraints at 6.8 and 16.7 ms, respectively.

## Results

**Figure 5:**
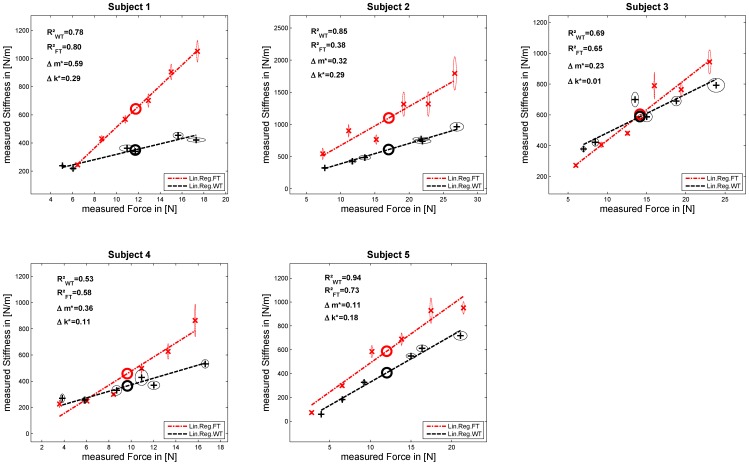
Results Experiment 1. Subjects doing the WT first without a cuff.

**Figure 6:**
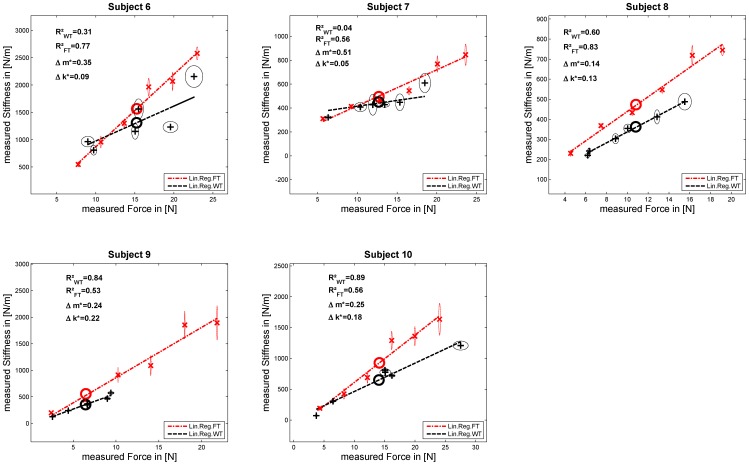
Results Experiment 2. Subjects doing the FT first without a cuff.

**Figure 7:**
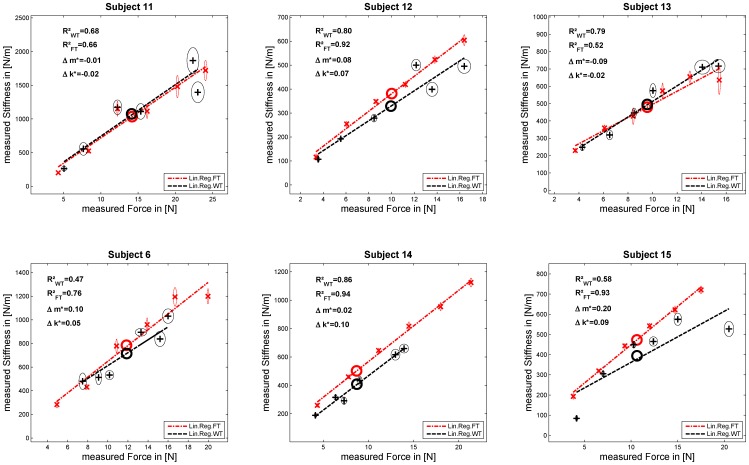
Results Experiment 3. Subjects who performed the experiment with the wrist cuff (WT first: top row, FT first: bottom row).

Fig. 5, Fig. 6, and Fig. 7 show the results of the experiments for each subject. Each graph depicts the measured stiffness for FT in red and WT in black and their linear regressions as dashed lines. For each force and weight level, the mean values and their SEM in force and stiffness and the mean in force and stiffness used for testing 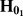 are plotted as circles. Additionally, the related 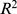 coefficient (values of 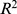 close to 1 denote a near-perfect linear regression) for a linear assumption between FT and WT and the two normalized mean inter-subject ratios of stiffness 
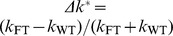
 and linear regression slopes 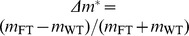 are depicted. Furthermore, Tables 1 and 2 list the results of the measured stiffnesses and the linear regressions between force and stiffness for each subject in both tasks. Based on these data and regression fits we performed statistical tests of the previously conjectured two hypotheses. For testing these, a fixed level of significance was chosen as 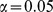 for all tests.

**Table 1:** Testing 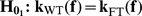.

group	subject	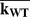 [N/m]	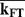 [N/m]	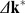	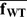 [N]	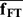 [N]	No. WT	No. FT	F-test [%]
E1	S1	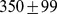	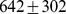	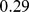	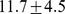	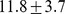	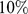	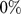	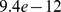 1
E1	S2	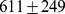	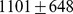	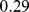	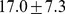	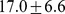	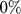	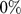	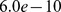 1
E1	S3	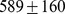	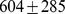	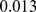	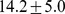	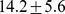	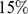	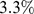	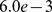 1
E1	S4	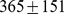	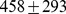	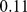	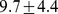	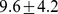	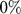	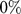	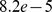 1
E1	S5	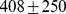	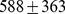	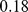	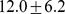	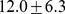	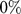	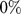	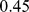 1
E2	S6-1	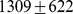	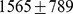	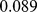	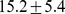	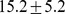	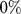	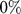	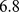
E2	S7	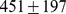	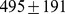	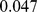	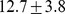	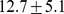	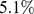	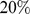	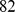
E2	S8	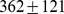	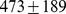	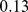	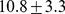	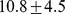	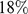	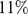	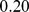 1
E2	S9	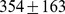	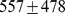	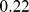	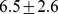	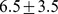	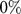	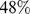	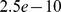 1
E2	S10	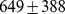	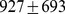	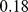	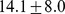	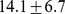	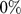	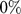	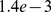 1
E3	S11	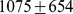	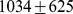	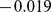	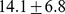	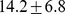	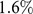	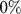	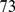
E3	S12	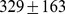	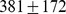	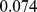	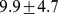	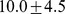	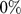	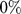	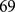
E3	S13	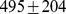	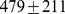	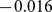	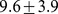	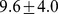	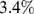	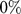	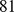
E3	S6-2	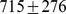	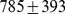	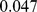	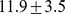	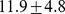	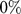	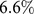	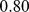 1
E3	S14	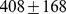	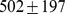	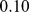	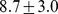	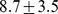	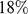	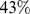	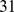
E3	S15	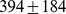	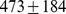	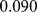	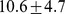	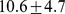	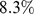	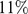	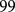

1
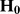
of the F-test (Variances are equal) is rejected for probability values less than 5%.

The mean and standard deviation of stiffness of both tasks, their normalized difference 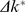, the percentage of data discarded for this tests and the result of the F-test are listed. Data were discarded such that mean in force of both data sets align.

**Table 2:** Linear regression and Mandels test for linearity.

group	subject	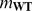	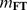	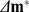	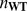	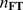	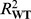	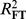	M-test WT	M-test FT
E1	S1	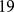	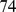	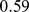	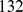	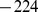	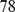	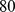	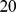	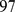
E1	S2	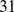	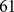	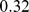	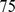	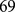	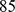	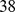	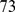	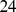
E1	S3	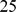	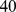	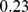	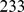	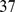	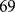	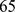	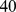	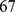
E1	S4	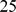	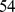	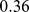	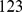	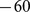	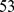	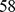	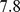	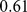 1
E1	S5	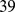	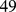	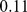	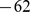	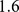	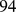	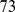	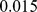 1	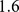 1
E2	S6-1	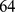	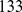	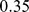	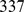	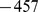	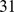	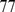	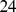	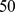
E2	S7	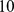	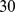	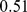	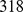	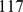	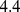	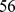	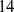	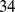
E2	S8	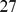	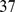	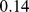	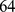	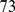	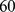	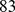	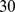	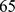
E2	S9	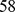	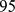	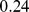	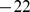	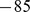	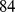	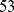	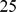	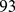
E2	S10	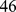	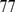	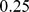	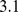	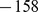	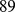	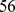	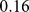 1	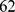
E3	S11	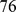	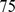	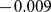	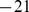	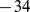	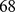	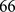	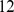	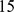
E3	S12	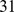	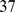	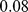	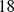	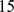	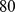	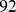	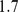 1	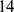
E3	S13	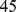	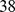	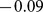	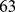	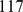	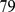	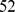	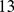	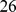
E3	S6-2	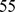	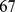	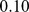	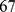	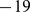	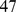	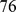	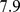	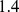 1
E3	S14	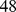	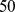	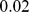	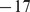	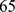	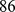	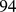	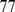	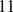
E3	S15	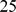	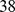	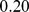	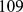	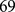	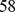	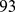	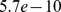 1	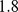 1

1 For probability values less than 5% it is rejected that a linear relation is as good as a quadratic.

Slope 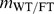 [1/m], their normalized values 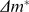, offset 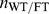 [N/m], the related 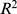 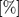 coefficient for a linear model and the results of the M-test in 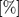 are listed.

### Results main experiment—groups E1 and E2

The subjects of groups E1 and E2 were asked to do either FT or WT first with an unconstrained wrist.


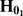
: **Equal stiffness for equal grip force (** 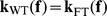 **)**

For each subject we tested whether the same stiffness was generated in each task, on average across all grip forces. Since the ranges of grip force differed considerably between the two tasks—especially for subjects doing the FT first (see, e.g., subject S9 in Fig. 6)—we algorithmically adjusted the datasets on a subject-by-subject basis by discarding trials with the highest or lowest grip-force values so as to align the mean grip forces before perturbation in WT vs. FT. We then compared the average stiffness levels. These data were only discarded for testing 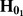. Note that this data adaptation had no qualitative effect on the results (rejection of tested hypothesis or not). Table 1 summarizes the results for each subject in detail; the mean values in force and stiffness are additionally depicted in Fig. 5–6. We tested whether grip stiffness differed for the two tasks by testing if their difference was significantly different from zero, on average across subjects, by performing Student's dependent paired t-test on mean inter-subject stiffness 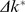, normalized by the sum of 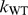 and 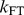 for each subject, for groups E1 and E2. These results (Table 3) indicate that mean stiffness differed significantly (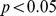) with higher stiffness measured in FT, regardless of which task was performed first by each subject (see Figs. 5 and 6). Furthermore, Table 1 includes results of an F-test for testing if the variances in intra-subject stiffness of both tasks were equal. The results provides evidence that, for all subjects of groups E1 and E2 excluding subject S6-1 and S7, it can be rejected that the variances in intra-subject stiffness were equal (Table 1), even if the tested standard variation was calculated across all data.

**Table 3:** Testing 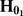.

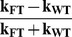	E1	E2	E1+E2
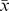	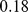	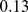	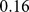
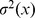	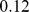	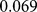	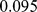
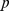 (two-tailed)	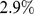 1	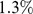 1	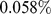 1

1
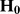
is rejected for probability values less than 5% (paired t-test).

Whereby the two stiffnesses Eqs. (1) and (2) are equal 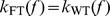 across subjects. The mean normalized difference of the two stiffnesses 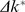 and its standard deviation (inter-subject variability). Data were discarded such that mean in force of both data sets align.


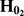
: **Equal grip-stiffness/grip-force slopes (** 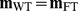 **) and offsets (** 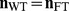 **)**

For each subject and task we did a linear regression between force and stiffness and calculated the slope and offset of the resulting regression (see Table 2 for details). We then tested whether the parameters of the grip-stiffness/grip-force regressions differed between the two tasks, on average across subjects via a dependent paired t-test using the normalized difference of the slopes 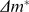 and the mean of the offsets (see Table 4). The results provide evidence that, in general, the slopes in FT differed significantly from WT (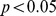) with higher slopes in FT, regardless of which task was performed first by each subject. Furthermore, it cannot be rejected that the mean offsets in group E1 or group E2 were equal; conversely, this can be rejected when data from the two groups were combined (E1+E2).

**Table 4:** Testing 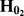

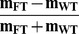	E1	E2	E1+E2	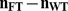	E1	E2	E1+E2
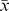	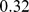	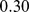	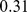	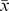	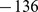	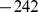	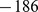
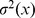	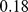	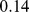	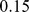	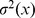	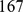	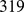	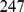
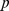 (two-tailed)	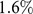 1	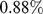 1	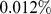 1	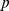 (two-tailed)	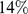	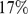	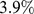 1

1
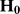
is rejected for probability values less than 5% (paired t-test).

Whereby the two slopes and offsets in Eqs. (1) and (2) are equal 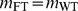 and 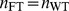 across subjects (inter-subject variability). Left table: The mean normalized difference of the two slopes 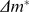 and its standard deviation. Left table: The mean difference of the two offsets and its standard deviation. No data were discarded.

What can we conclude from the above? When holding the weight in the hand, higher muscle activation was required for WT in order to counteract the vertical load. Furthermore, to stabilize the wrist against this vertical load, antagonistic pairs of muscles will have been activated. One might expect a higher measured stiffnesses in comparison to FT, in which no additional weight must be supported. But the opposite was measured: by increasing the load, the stiffness decreased at constant grip force.

One could argue that the higher stiffness in the FT was required to accurately hold a certain force level using cocontraction, while for the WT it was not, because there no visual feedback of force was presented. As we discussed in section “sec:Model,” using cocontraction will affect the offset of the stiffness/force relation. Because the results of testing 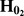 provides evidence that the regressed offsets of the two tasks were different, we were interested to know if each of them differed from zero. We found that it can be rejected for the WT across both groups E1 and E2 that the offset listed in Table 2 is equal to the origin (two-tailed t-test; 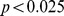), but not for the FT.

We furthermore looked at the correlation between slope and mean intra-subject stiffness across all subjects (no data were discarded; see Table 5). The results indicate that there is a significant correlation between mean stiffness and slope for the FT, which further argues for a stiffness/force relation going through the origin for the FT. As a corollary, the results are consistent with the finding that the stiffness/force curve of a single muscle most likely goes through the origin [20]. Note that the offset of measured stiffness/force characteristic of the antagonistic system at zero net force is not precisely zero because of the *passive* stiffness of surrounding tissues and ligaments in the arm and hand.

**Table 5:** Correlation 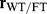 between slope and mean intra-subject stiffness and its probability 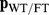 in % for groups E1 and E2.

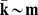	E1	E2	E1+E2
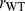	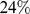	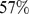	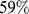
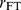	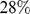	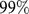	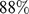
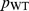	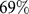	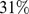	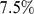
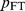	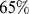	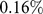 1	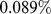 1

1 The correlation is significant.

For probability values less than 5% the correlation is significant. No data were discarded.

### Results experiments with fixed wrist—group E3

Given that we found both higher grip stiffness and a higher grip-stiffness/grip-force slope for FT together with an offset in the FT not different from zero and an offset significantly larger than zero for the WT, our results argue strongly for a change in the kinematics as the predominately underlying mechanism rather than a change of cocontraction (see section “sec:Model”). To further test this hypothesis, subjects in group E3 performed the two tasks with the wrist held in a constant position by a rigid cuff in order to minimize the influence of a change in the kinematics. The cuff used here was made of a thermoplast with a steel plate parallel to the arm axis in order to maximize its stiffness; but still the wrist could be bent within small ranges. In order to prevent subjects from moving their wrist, we additionally rested the arm and hand on a table.

We compared the average stiffness across all grip-force levels, the slope of the grip-stiffness/grip-force relationship and their correlation, the results of which are shown in Table 6. One can see that grip stiffness – grip force relationship, i.e. the slopes and the offsets, differed much less between the two tasks when the wrist was stabilized. Nevertheless, grip stiffness was still higher for FT versus WT. Furthermore, there was a highly significant correlation between mean intra-subject stiffness and the slope of the stiffness/force curve for the WT (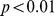) and FT (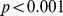). It cannot be rejected for either task that the mean offset across subjects was equal to zero. Further, the results on testing equal variances in intra-subject stiffness using the F-test provides evidence that, for all subjects of group E3 except 6-2, it cannot be rejected that these variances were equal.

**Table 6:** Results of group E3 when the wrist was held in a constant posture by a cuff.

E3	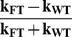	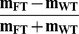	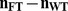	E3	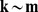
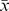	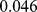	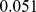	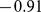	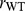	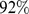
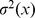	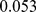	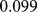	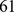	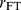	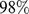
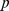 (two-tailed)	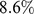	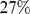	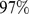	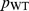	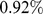 2
				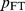	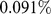 2

1
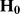
that 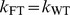, 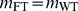 and 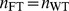 is rejected for probability values less than 5% (paired t-test), respectively.

2 For probability values less than 5% the correlation is significant.

Left table: dependent paired t-test of greater inter-subject grip stiffness, grip-stiffness/grip-force slope and offset for FT versus WT. Right table: Correlation between slope and mean intra-subject stiffness and its probability in %. No data were discarded.

### About linearity between force and stiffness

As we initially stated we found a strong linear correlation between force and stiffness during the FT. To close out our description of section “sec:Results” we will further test if linearity was indeed obtained and if it changed between tasks. We used Mandel's technique to test whether a linear or a quadratic model provides a significantly better fit for the relationship between grip stiffness and grip force ([21], p. 165ff). The test compares the standard deviations of the residuals
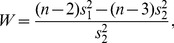
(18)where 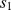 and 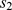 are the residual standard deviations of a linear and quadratic fit and 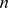 the sample size. 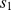 and 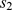 are computed as
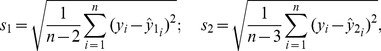
(19)where 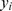 are the measured and 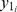, 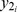 the fitted values. If the linear model is a correct assumption, 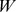 will be close to 1; if the quadratic model is a better assumption, the numerator will tend to be larger than the denominator. The Mandel test uses the F-distribution to test for significance: If 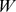 is less than or equal to the value of the 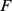-distribution 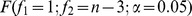, it can be rejected that a quadratic model provides a considerably better fit to the measured values (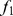 is the number of degrees of freedom of the numerator; 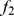 of the denominator).

The results of the Mandel test are listed in Table 2. Note that we also corrected the data for non-normality (see [22], p.78ff for details), because the F-test is very sensitive to non-normally distributed data. Since the results of the Mandel test are identical (rejection or not), we will refrain from a detailed explanation for correction of non-normality. The results listed in Table 2 provide evidence that a linear relationship captures the underlying relationship to a reasonable degree for most of the subjects and tasks, consistent with our findings in [9].

Note the difference between 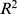 and the Mandel test for linearity. 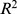 indicates the percentage of the variance which can be explained by a (cq.) linear model. Thus, by implication 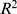 of a quadratic model is never worse than that of a linear one. The Mandel test compares the difference of both model residuals by taking also the statistical degrees of freedom into account and indicates whether the difference is significant. E.g., the amount of data that can be explained by a linear model for the WT of subject S5 is not bad (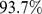) and indicates a linear relation, but using a quadratic model is significantly better (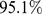).

## Discussion and Conclusions

The main result of these experiments is that grip stiffness is regulated independently from grip force, at least to some extent. The conventional assumption that stiffness increases linearly with applied force did hold in all of our experimental conditions, but the parameters of that linear relationship varied according to the task. Mean grip stiffness was considerably higher in FT than in WT, for all subjects of the groups E1 and E2, without any significant differences between early and late trials of single subjects, in much the same way that average grip force varied between static and dynamic grasping of different weights [23], regardless of which task was performed first by each subject. But the slope of the grip stiffness/grip force relationship was also higher for FT. Furthermore, WT stiffness was higher for lower grip forces (compare subjects S1, S3, S4, S6-1, S7 and S10). This is confirmed by the finding that over all subjects of both groups the offsets were significantly higher in the WT (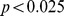; one-tailed dependent paired t-test; find the corresponding mean and standard deviation in Table 4). Additionally, the WT offsets were significantly higher than zero, while the FT offsets were not. Together with a strong correlation between slope and mean intra-subject stiffness for the FT, these results portend to a change of finger/wrist configuration as the predominant mechanism underlying this change in the stiffness/force relationship, as opposed to a change in the level of cocontraction of antagonistic muscles of the fingers. We tested this hypothesis with a new set of subjects with their wrist fixed by a cuff and rested on a pedestal so as to maintain the same posture at the wrist. We found that both curves matched in terms of stiffness, slope, and offset and for both tasks a strong correlation between mean intra-subject stiffness and slope was observed. Furthermore, we found variances in intra-subject stiffness matched as well, which also argues in favor of data coming from the same population and thus for similar experimental conditions in both tasks. However, even if not for subjects S11 and S13, the measured mean stiffnesses in the FT were still somewhat higher for the group (see Table 6).

The limitations of our experimental conditions in E3 had to be mentioned as well: fixating the wrist and resting it on a pedestal in order to prevent the wrist from bending reduced activation in WT, possibly leading to lower WT stiffnesses. As we initially explained in section “sec:Model”, a change in kinematics possibly predominates effects of cocontraction on the stiffness/force characteristics. Thus, from measurements done within E3 it cannot be excluded, that the difference found in E1 and E2 is a combination of a change in kinematics *and* cocontraction, with subjects using both strategies in the WT simultaneously.

Furthermore, it should be acknowledged that our results are only valid for expanding objects and thus for the measured stiffness to force characteristics of corresponding musculotendon structure. The measured stiffness to a contracting object might be different and therefore characterizing the reaction as a linear stiffness might not be appropriate. In [10] Van Doren measured grip stiffness by measuring exerted forces of a contracting and expanding handle and used the subtraction of respective forces of both for calculating grip stiffness. But even if the meaning of stiffness measured in this work is different to our estimation, and includes information of an expanding and contracting object (and of reflexes as well), the authors still found a monotonic increase of stiffness with grip force.

Comparing the slopes and the mean stiffness between subjects, one can see that they differed considerably. Some of these large differences in inter-subject stiffness could be explained by a difference in grip force, but certainly not everything. Furthermore, for groups E1 and E2, some of the inter-subject variability can be explained by different wrist positions of the subjects. But even within group E3, where the wrist was fixed in one position, the stiffnesses differed considerably between subjects. Measuring planar human arm stiffness, Mussa-Ivaldi [24] reported that qualitative measures such as *shape* and *orientation* of a stiffness ellipse measured at the endpoint are similar over different subjects for different postures, but the quantitative measure *size* is not, and even varies considerably for identical subjects measured on different days.

The force ranges differed considerably between the two tasks, especially for subjects doing FT first. Since we wanted to exclude the influence of which task is done first, we let the subjects of this group only lift each weight once before the FT in order to avoid learning. This leads to a data set of 6 different force levels, while for subjects doing WT first a data set of 60 data points was used to calculate the force levels. Thus, the force ranges between both tasks differed more for the group doing FT first, leading to a larger force range of FT. However, we expect the influence of this difference to be small. Remember that to test 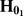 we only compared data within equal force ranges by discarding extreme values (6.6%) of the data set.

A number of studies have already demonstrated how the finger [25] and wrist [16] can affect fingertip forces and stiffness in the human hand and the idea that one might adjust the configuration of a redundant multi-joint linkage to optimize impedance with respect to the task or to the environment [26], [27] has also been proposed. The question which remains to be clarified here is: Are the found differences between the two tasks actively controlled by the CNS? And if yes, why should subjects minimize the influence of the wrist in an FT where they get a visually-presented feedback about variations of the actual grip force?

To maximize the efficacy of the visually-guided control loop, one could reasonably strive to minimize the latency between commanded changes in muscle activations and the actual changes of grip force applied at the fingertips. As muscles are constrained by activation dynamics, there is a theoretical limit to the rate of change of muscle force 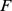 with respect to time, 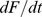, that a given muscle can produce. The rate of change of force measured at the fingertip would be modulated by the same Jacobian that governs the relationship between muscle force and finger force, and between muscle stiffness and finger stiffness, i.e., 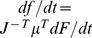. Thus, by maximizing the norm of 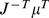, one maximizes the ability to rapidly effectuate a change in grip force in response to a visually presented force error. From this point of view, the modulation of grip stiffness observed in our experiments is simply a corollary of the real optimization, that of maximizing responsivity to a visual command, rather than an optimization of grip impedance *per se*, to the differences in mechanical constraints between FT and WT. On the other hand, the signal-dependent noise of the corresponding sensors (viz. the Golgi tendon organs) increases with their activation, and will therefore increase its effect on fingertip force. Conclusively, we can only clarify to some extent and not with significance if the found difference is actively optimized or just passively caused by a bent wrist.

All in all, of course static grip stiffness is linearly related to grip force, caused by the exponential stiffness of tendon tissue. But by changing the kinematics of our grip—and thus just changing the force transfer function from muscle to finger—one can actively change the increase of stiffness with force by flexing our wrist and thus change the stability of the grip. In that it is highly relevant that, in our experiments, low stiffness values were obtained when holding objects of different weights rather than exerting a predefined force—a natural task, which we likely have learned to solve at minimal cost.
