# Correction: Task Dependency of Grip Stiffness—A Study of Human Grip Force and Grip Stiffness Dependency during Two Different Tasks with Same Grip Forces

**DOI:** 10.1371/annotation/a78174f1-8079-4ec1-ad48-b10dc2d29062

**Published:** 2014-01-17

**Authors:** Hannes Höppner, Joseph McIntyre, Patrick van der Smagt

The images and legends for Figures 1, 2, and 3 are switched. 

Please view Figure 1 here: 

**Figure:**
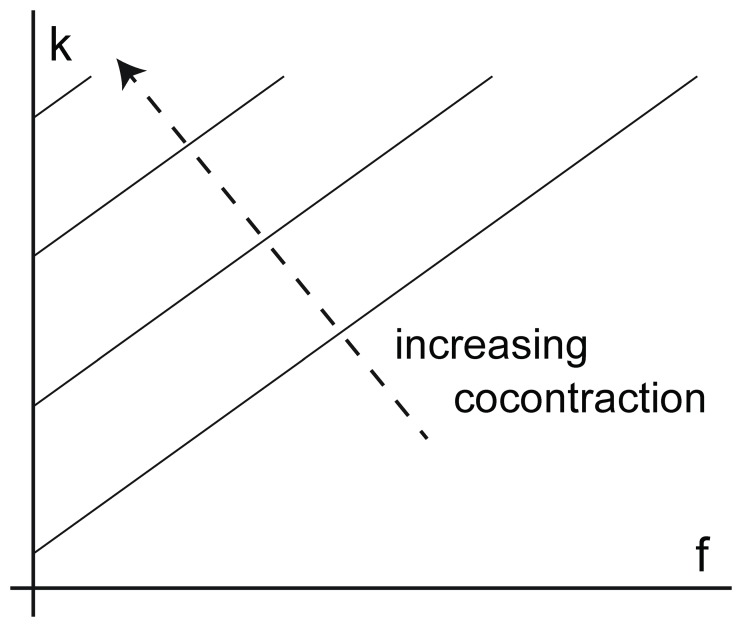


The Figure 1 legend should read: Figure 1. Stiffness change due to cocontraction. Increasing the cocontraction of the corresponding muscles increases the offset in the stiffness/force relationship.

Please view Figure 2 here: 

**Figure:**
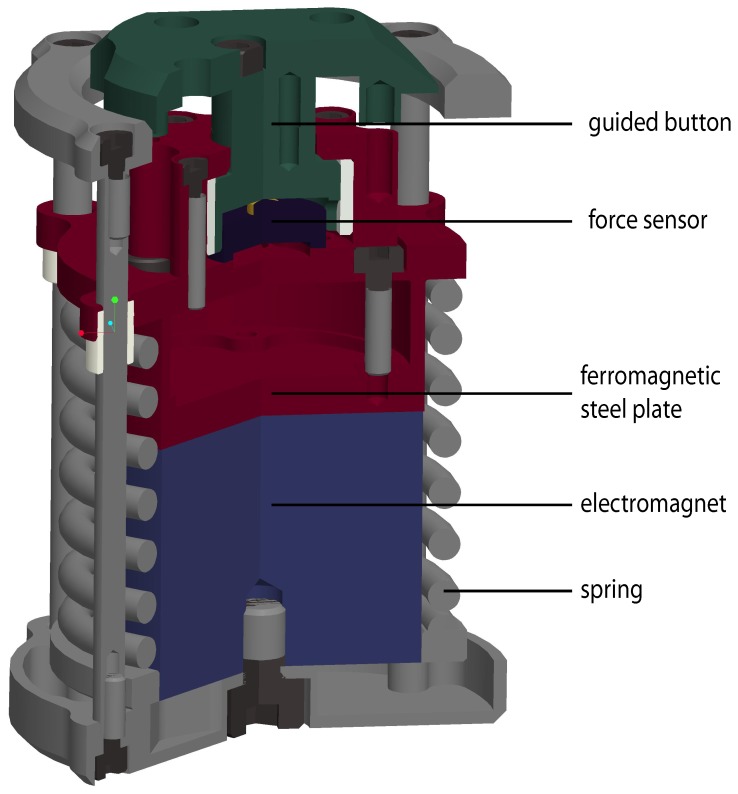


The Figure 2 legend should read: Figure 2. Cross sectional view of the Grasp Perturbator.

Please view Figure 3 here: 

**Figure:**
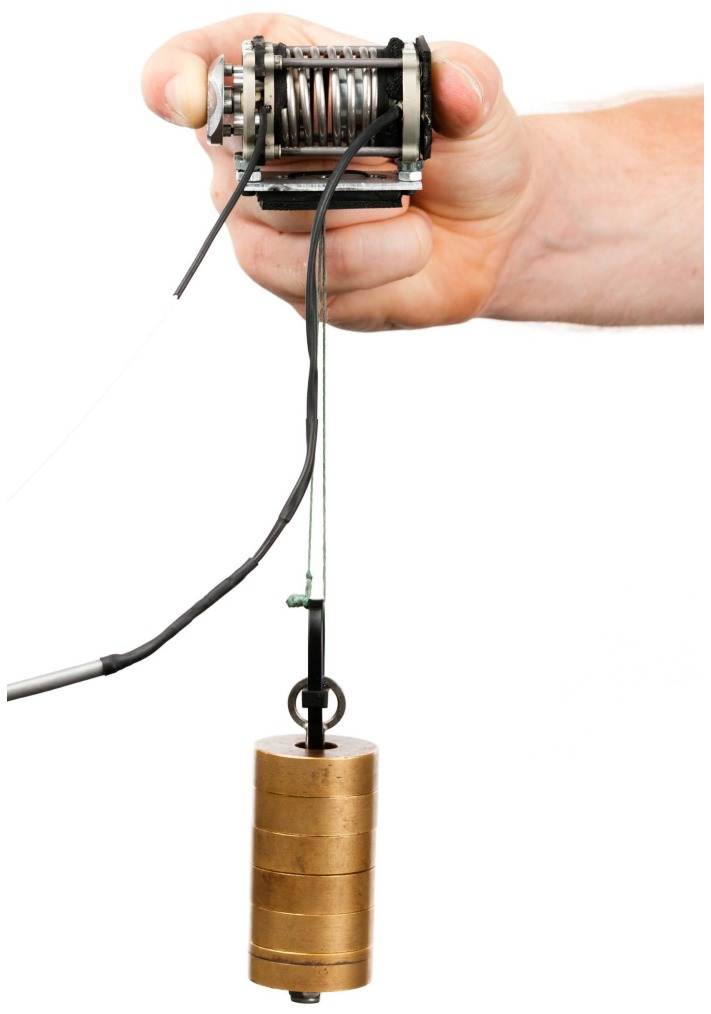


The Figure 3 legend should read: Figure 3. Grasp Perturbator held in a pinch grasp with attached weights.

Various formatting errors occurred throughout the tables of this article. Please view the following tables here:

Table 1: 

**Figure:**
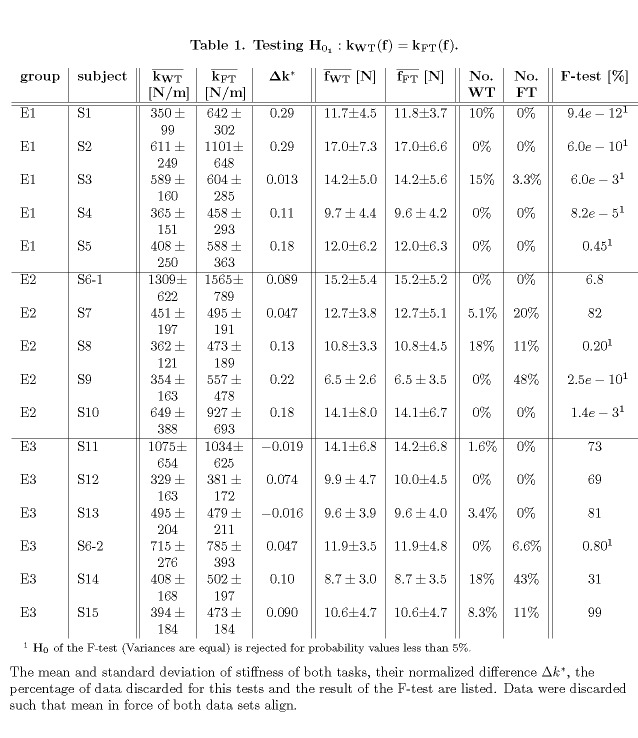


Table 2: 

**Figure:**
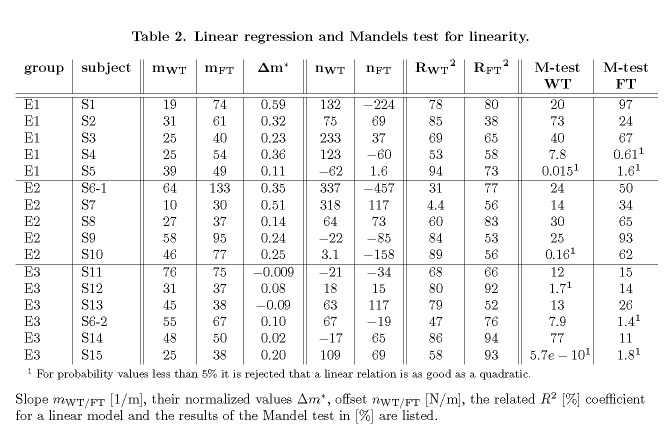


Table 3: 

**Figure:**
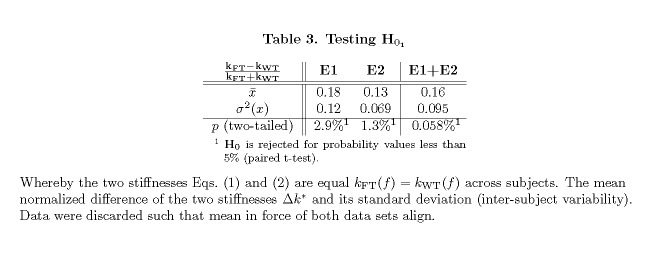


Table 4: 

**Figure:**
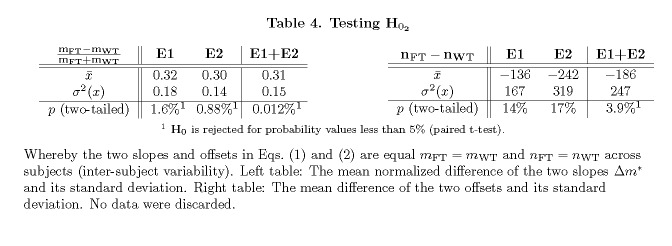


Table 5: 

**Figure:**
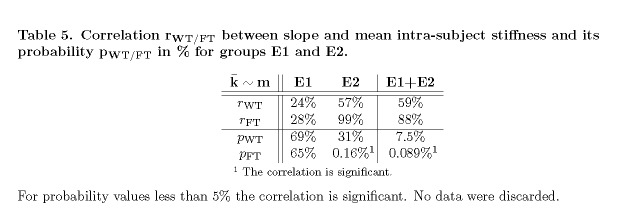


Table 6: 

**Figure:**
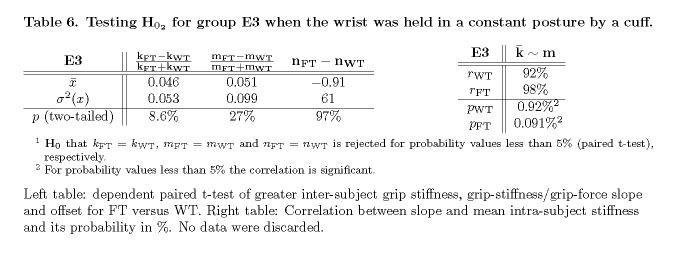


Here is an explanation for the acronyms used throughout the text:

SEM – standard error of mean

M-test – Mandel test

CNS – central nervous system

CSE – coefficient of standard error

All instances of "sec:Model" should read "General Model of the fingers"

All instances of "sec:Results" should read "Results"

